# Spray-Induced Silencing of Pathogenicity Gene *MoDES1 via* Exogenous Double-Stranded RNA Can Confer Partial Resistance Against Fungal Blast in Rice

**DOI:** 10.3389/fpls.2021.733129

**Published:** 2021-11-26

**Authors:** Atrayee Sarkar, Subhankar Roy-Barman

**Affiliations:** Department of Biotechnology, National Institute of Technology, Durgapur, India

**Keywords:** rice, fungal blast, *MoDES1*, double-stranded RNA (dsRNA), spray induced gene silencing (SIGS), disease resistance, RNA-based crop protection

## Abstract

Over the past years, RNA interference (RNAi) has been used as a promising combat strategy against a wide range of pests and pathogens in ensuring global food security. It involves the induction of highly specific posttranscriptional regulation of target essential genes from an organism, *via* the application of precursor long, non-coding double-stranded RNA (dsRNA) molecules that share sequence-complementarity with the mRNAs of the targets. Fungal blast disease caused by *Magnaporthe oryzae* is one of the most deadly diseases of rice and wheat incurring huge losses in global crop yield. To date, the host-induced gene silencing (HIGS) and virus-induced gene silencing (VIGS) aspects of RNAi have been successfully exploited in developing resistance against *M. oryzae* in rice. Spray-induced gene silencing (SIGS) is a current, potential, non-transformative, and environment-friendly pest and pathogen management strategy, where naked or nanomaterial-bound dsRNA are sprayed on leaves to cause selective knockdown of pathogenicity genes. Although it relies on the ability of fungal pathogens to uptake sprayed RNA, its efficiency varies largely across phytopathogens and their genes, targeted for silencing. Here, we report a transient dsRNA supplementation system for the targeted knockdown of *MoDES1*, a host-defense suppressor pathogenicity gene from *M. oryzae*. We validate the feasibility of *in vivo* SIGS and post-uptake transfer of RNA signals to distal plant parts in rice-*M. oryzae* pathosystem through a *GFP*-based reporter system. A protocol for efficient silencing *via* direct foliar spray of naked dsRNA was optimized. As proof-of-concept, we demonstrate the phenotypic impacts of *in vitro* ds*DES1* treatment on growth, conidiation, ROS-scavenging ability, and pathogenic potential of *M. oryzae*. Furthermore, our extrapolatory ds*DES1* spray experiments on wounded leaves and whole rice plants indicate resultant silencing of *MoDES1* that conferred significant resistance against the fungal blast disease. The evaluation of primary and secondary host defense responses provides evidence supporting the notion that spray of sequence-specific dsRNA on wounded leaf tissue can cause systemic and sustained silencing of a *M. oryzae* target gene. For the first time, we establish a transgene-free SIGS approach as a promising crop protection strategy against the notorious rice-blast fungus.

## Introduction

Plants, like all other organisms, have to survive in an environment where numerous microorganisms coexist. The interaction between plant and microbes is very specific when it comes to disease development. In order to evade pathogenic attacks, plants have developed an immune system that comprises a network of defense systems and molecules. The fundamental strategy to survive among a myriad of pathogens is the fast recognition of an invading pathogen, followed by the rapid induction of a multifaceted defense response. In plants, the first line of defense involves the perception of some signature molecular motifs specific to different classes of pathogens, which are known as pathogen-associated molecular patterns (PAMPs), recognized by some transmembrane pattern recognition receptor (PRR) proteins (Medzhitov and Janeway, 1997). Once the ligand-binding ecto-domain of PRRs get activated by other trans-membrane signaling adapters (Zipfel et al., 2006), the identification of the pathogen results in systemic acquired resistance (SAR) in plants (Mishina and Zeier, 2007). SAR is an integral part of PAMP-triggered immunity (PTI) and is known to confer durable resistance to the host against a broad spectrum of pathogens (Durrant and Dong, 2004). It induces physiological and molecular adaptations, namely, alteration in flux of ions such as Ca^2+^, H^+^, and Cl, across the plasma membrane (Garcia-Brugger et al., 2006), and the expression of WRKY transcription factors (Pandey and Somssich, 2009) that sometimes control reactive oxygen species (ROS) burst and salicylic acid (SA) production (Takabatake et al., 2007; Du et al., 2009). SA and other hormones such as jasmonic acid (JA) and ethylene (ET), are classically known to be involved in plant defense *via* the induction of pathogenesis-related (PR) genes against a wide range of pathogens (Bari and Jones, 2009). However, there is constant coevolution happening in the front of both pathogens and plants (Jones and Dangl, 2006). In order to overcome PTI successfully, some pathogens undergo modification in their genome and produce effectors. On recognition of such hypervariable molecules, effector-triggered immunity (ETI) develops in plants. Often, it encompasses specific “gene to gene” interaction between the effectors or avirulence (Avr) factors and R-proteins that the plants synthesize to counter such effector molecules (van der Biezen and Jones, 1998; Chisholm et al., 2006). ETI, which is characterized by cell death, lignification, ROS formation, and hypersensitive response (HR), is specific and robust (Nürnberger et al., 2004). Together, HR and the synthesis of toxic ROS are essential markers of plant immunity, as they influence other responses such as lignin and callose deposition, and phytoalexin synthesis (van Loon et al., 1998; Van Loon and Van Strien, 1999).

Rice (*Oryza sativa*) is a major agricultural crop consumed by over 50% of world population. Around 10–30% of its annual yields that could feed around 60 million people get destroyed by the fungus *M. oryzae* (Pennisi, 2010). Besides rice, this fungus also causes blast disease in another staple crop, wheat, where it may destroy the whole crop produce. Having made its position among the top 10 fungal diseases that infect plants (Dean et al., 2012), it continues to pose a major threat to global food production. Besides the use of fungicides and pesticides, developing genetic resistance against plant diseases by breeding-based crop improvement programs is one of the most popular and acceptable approaches to address food security. Although they are often achieved by traditional breeding, several other approaches involving genome editing and genetic modification have been in practice for imparting more durable resistance against the ever-evolving pathogens. The overexpression of defense-inducing factors such as PRRs, R genes, and even effectors (Wang et al., 2019), or the knockout of susceptibility (S) genes (Santillán Martínez et al., 2020) in host plants can provide a strong armament for arms race against its pathogen.

Highly unstable ROS-like peroxides and superoxides are known to be versatile players in disease development (Torres and Dangl, 2005; Shetty et al., 2007; Kou et al., 2019). In the case of the fungal blast disease, while *Magnaporthe*-NADPH oxidase-mediated ROS generation helps it in causing disease (Egan et al., 2007), ROS burst by NADPH-oxidase of invaded rice cells can prevent fungal colonization and induce resistance *via* callose formation and the expression of defense-related genes (Torres et al., 2006). *M. oryzae* penetrates rice cells *via* a special melanized appressorium that is formed at the tip of a germinated asexual spore or conidium. The appressoria form a penetration peg that differentiates inside the primary host cell to form invasive hyphae (Kankanala et al., 2007). One of the major pathways activated in rice cell in this stage in order to counter against fungal ROS is the *Os* chitin elicitor-binding protein (CEBIP)/*Os* chitin elicitor receptor kinase 1 (CERK1)-*Os* guanine nucleotide exchange factor(RACGEF1)-*Os*RAC1 module that is a pivotal component of the defensome involved specifically in chitin-mediated PTI (Akamatsu et al., 2013). *Os*CEBIP is a rice receptor-like protein without an intracellular kinase domain that recognizes fungal chitin. Upon binding to chitin directly, it dimerizes, and the CEBIP dimer interacts with the PRR *Os*CERK1 (Kaku et al., 2006; Shimizu et al., 2010). The receptor complex then activates *Os*RACGEF1 by phosphorylation. Therefore, in response to chitin, *Os*RACGEF1 activates *OsRAC1*, which switches on rice immunity by transducing signals to various downstream signaling components: NADPH oxidase (Rboh) for ROS production, lignin synthesis (Kawasaki et al., 2006), induction of cell death, PR-genes, and phytoalexins (Dang et al., 2013).

One of the ways by which *M. oryzae* secures its successful pathogenesis is by scavenging these host NADPH oxidase-generated ROS. Enzymes, such as peroxidases (Mir et al., 2015), redox-sensitive transcription factors (Guo et al., 2011; Qi et al., 2012), and pathogenicity factors that influence extracellular enzyme activity *via* pleiotropic changes, are central to the fungal defense against exogenous or host-derived ROS. Being a hemibiotroph, in a susceptible plant, *M. oryzae* suppresses the metabolic pathways associated with ROS formation after incipient ROS-burst *via* effectors and defense suppressors (Samalova et al., 2014). *MoDES1* is one such innate defense suppressor of rice that is important in detoxification of plant-driven ROS, and crucial for pathogenesis. It gets triggered in response to initial PTI-mediated ROS burst and is the encounter strategy of the pathogen against its host to suppress downstream defense-associated signaling, such as further ROS generation, HR, and SAR (Chi et al., 2009). *MoDES1* mutants showed difficulty in fungal penetration and colonization, and ROS-sensitivity because of reduction in extracellular detoxifying enzyme activity (Chi et al., 2009; Kou et al., 2019).

Apart from traditional crop breeding approaches, one of the key strategies of developing durable resistance against a pathogen on field, is the targeted *in planta* knockdown of one or more pathogenicity factor(s). RNA interference (RNAi) technology can specifically silence a target gene *via* a dsRNA (double-stranded RNA) molecule sharing sequence complementarity with its mRNA (Fire et al., 1998; Wilson and Doudna, 2013). The RNAi tool has been used successfully in developing resistance against pests, pathogens, and viruses (Zotti et al., 2017). Nowadays, two major aspects of using RNAi in crop improvement are transgene-mediated HIGS and exogenous dsRNA spray-induced gene silencing (SIGS), with the central principle being the same. The already proven efficient HIGS (Baulcombe, 2015) is based on the ability of filamentous fungi to take RNA species (double-stranded and siRNAs) from host plants while drawing nutrition through haustoria. A successful demonstration of effective assimilation and processing of dsRNAs has been reported in several plant pathogenic fungi such as *Botrytis cinerea* (Wang et al., 2016), *Fusarium graminerium* (Koch et al., 2016; Baldwin et al., 2018), *Fusarium asiaticum* and *Fusarium* sp. (Song et al., 2018), *Magnaporthe oryzae* (Zhu et al., 2017; Guo et al., 2019), and *Puccinia striiformis* fsp. *tritici* (Qi et al., 2018), *via* their transformed hairpin-loop-expressing host. On the contrary, SIGS is a relatively new and emerging “non-transgenic” crop-protection strategy that looks promising. The spray application of dsRNA on plants has been able to suppress endogenous genes and transgenes in a target plant (Dubrovina and Kiselev, 2019), and has efficiently silenced genes among several insects, pests, and pathogens (Morozov et al., 2019). The efficacy of SIGS in suppressing specific genes in a pathogen lies not only on the machinery of RNAi but also on the cross-kingdom movement and exchange of RNAs between the plant and the interacting pathogen (Wang and Dean, 2020). The extracellular vesicles of a plant play a crucial role in transporting sRNA cargo across the plant-fungus interface, after plant dicers process the exogenously sprayed dsRNA, thereby leading to the homology-based degradation of fungal target transcripts (Cai et al., 2021). Since the response of different host-pathosystems toward the phenomenon may vary, it is essential to assess the efficiency of SIGS in curbing a particular disease. In *M, oryzae*, while a few reports have been published about HIGS, the SIGS approach is relatively less explored. The silencing of a specific target gene, *MoAP1*, achieved by the spraying of artificial siRNAs (asiRNAs) on rice leaves has been reported to reduce disease symptoms caused by *M, oryzae*. Although that was a proof-of-concept experiment for HIGS (Guo et al., 2019), no dedicated study on SIGS has been conducted on the fungal blast-rice pathosystem to date.

In this study, we tried to address the possibility of utilizing SIGS on *M. oryzae* to reduce knowledge gap and generate more insights into the process. Considering recent reports on different pathosystems into consideration, we hypothesized that SIGS might act as a promising transgene-free alternative to HIGS in controlling fungal blast disease. Unlike HIGS, which involves the stable expression of a hairpin construct in the host, SIGS can be a relatively simpler, less time-consuming, and robust strategy for selectively targeting pathogenicity factors, thereby conferring disease resistance. However, the efficiency of eukaryotic pathogens to uptake externally applied dsRNA generally varies, and the assertion of SIGS largely depends on that factor (Qiao et al., 2021). Therefore, to prove our hypothesis, SIGS was first demonstrated in the *M. oryzae*-rice pathosystem using a *green fluorescent protein* (*GFP*) reporter system. dsRNA uptake-efficiency and specificity, and its *in planta* impact were assessed in dsRNA-sprayed rice plants. The impact of spraying dsRNAs on both intact and wounded leaves was evaluated to better understand the sustenance of gene silencing through RNA signals. Furthermore, SIGS was performed to selectively target *MoDES1* in rice, and the status of rice defense response was analyzed. The results generated from this study indicate that SIGS can efficiently target a pathogenicity gene and render this pathogen less-suited for pathogenesis, and experimentally established that *MoDES1* could be a target candidate gene for the development of resistance against *M. oryzae* in other hosts. Our optimizations will also help in the potential utilization of this gene knockdown strategy more efficiently in the rice-*M. oryzae* pathosystem. To our knowledge, this will be the first study dedicated to *in planta* SIGS contributing to disease resistance in this model pathosystem. We hope that these findings will open leads toward transient RNAi-based research on this destructive pathogen and further exploration of the functional and mechanistic aspects of the lesser-known SIGS technology.

## Materials and Methods

### Fungal Strains, Rice Varieties, and Vector, and Their Growth Conditions

In this study, an Indian strain of *M. oryzae*, i.e., B157 (international race IC9) isolated from Hyderabad, and its cytoplasmic GFP-expressing transformant (*M. oryzaeGFP*) were grown on yeast extract glucose (YEG) media (Saha et al., 2020). The blast-susceptible indica rice cultivar- C0-43, was grown at 27°C under a 16:8 light/dark photoperiod. The binary vector pCAMBIA1302 was streaked onto Luria Bertani (Himedia, Mumbai, India) agar plates supplemented with 50 mg/L Kanamycin (Himedia, Mumbai, India) and incubated overnight at 37°C. Fungal growth assays were performed on a complete medium (CM) supplemented with or without dsRNA, at 28°C for 7 days. For conidiation, the fungi were grown in dark on YEG plates for 8–10 days. The fungal biomass was then scraped on 10 days after inoculation and homogenized into uniform slurry by vortexing. Spores were separated from the mycelial debris by filtering the slurry through sterile MiraCloth (Calbiochem, Darmstadt, Germany), and then centrifuged at 7,000 rpm for 7 min. The conidia were visualized and counted under a microscope using a hemocytometer (Neubauer, Marienfeld, Germany). The length and breadth of conidia were measured keeping a 20-μm scale reference using an built-in microscope camera. Microscopic examination of at least 50 conidia per replicate was done in at least three independent experiments.

### Isolation of Fungal Genomic DNA and Polymerase Chain Reaction

The fungal culture was grown in YEG broth by the inoculation of solid culture agar blocks. Fungal balls were filtered out of the 4-day-old culture, and mycelia were harvested, dried, and weighed before genomic DNA isolation was carried out (Dellaporta et al., 1983). Plasmid DNA was isolated using alkaline lysis method (Sambrook et al., 1989) from the fully grown culture. Each 20 μl PCR reaction was set up for 100 ng pure genomic DNA or plasmid DNA containing forward and reverse primers, deoxynucleotide triphosphates (dNTPs), 10 × reaction buffer, and 1.5 units of Taq DNA Polymerase (NEB, Ipswich, Massachusetts, United States). The PCR was carried out in a thermal cycler (Genetix, New Delhi, India), with standard PCR conditions, whereby the annealing temperatures were maintained at 52 (for *GFP*) and 54°C (for *MoDES1*) for 30 s per cycle. Resultant PCR products were used for *in vitro* transcription, as will be described later. The primers used are listed in Supplementary Table 2.

### Design and Synthesis of Double-Stranded RNA

**FIGURE 1:**
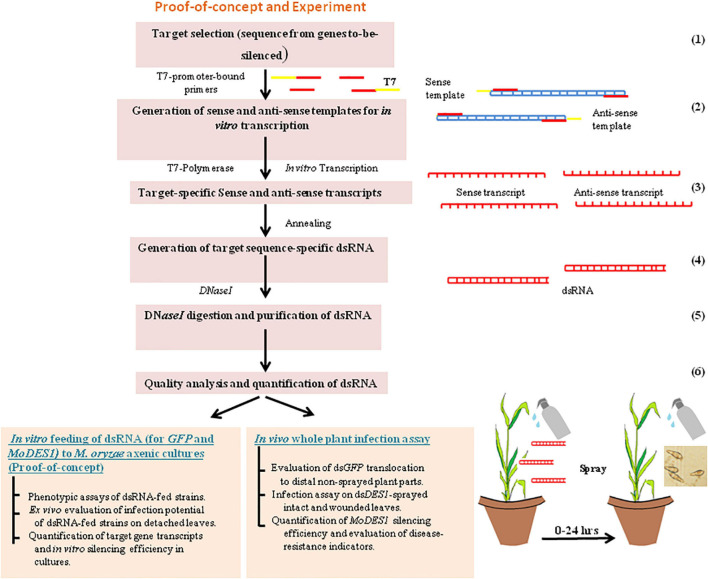
Schematic flow of work. Schematic flow diagram for *in vitro* synthesis of dsRNA, and proof-of-concept representing its *in vitro* feeding into *Magnaporthe oryzae* axenic cultures, *in vivo* spray-application on rice plant, and infection assay. Numbers on the right hand side indicate the steps involved in the process.

The sequence information of *GFP* [obtained from pCAMBIA-1302 (GenBank: AF234298.1)] and *MoDES1* (MGG_04163) was obtained from NCBI.^1^ Target regions were selected from their exonic regions, as indicated in Supplementary Figure 1. A target sequence homology of more than 19-mer with any other gene can render non-specificity to the precursor dsRNA. Hence, the target sequences were checked for homology across the whole genome assembly of the *M. oryzae*, *O. sativa* subsp. *indica*, *O. sativa* subsp. *japonica*, and *O. sativa* subsp. *javanica* groups using NCBI BLASTn. Homology was similarly checked between *MoDES1* target sequence and its counterparts coding for its homologs known from other closely related Ascomycetous fungi (Chi et al., 2009). Only members that showed a significant percentage sequence similarity of greater than 40% were considered for BLAST analysis with the *MoDES1* dsRNA sequence. After ensuring that there was no significant similarity among the interrogated sequences, the target regions (Supplementary Figure 1) were PCR-amplified from the *M. oryzaeGFP* genomic DNA using sense and anti-sense primers linked with a T7 promoter sequence at their 5′ ends (Supplementary Table 2). While the sense forward primer (FP) and the anti-sense reverse primer (RP) had a T7 promoter sequence attached to them, the sense RP and anti-sense FP did not have it. The PCR conditions have been discussed above, in the “genomic DNA isolation and PCR” section of “Materials and methods.” The amplicons were checked for band specificity, purified using Purelink PCR Purification Kit (Invitrogen, Waltham, MA, United States), and a further 1 μg each of individual amplicons were used as templates for T7 DNA-dependent RNA polymerase-based *in vitro* transcription. For this, TranscriptAid T7 High-Yield Transcription Kit (Invitrogen, Waltham, MA, United States) was used following the instructions of the manufacturer. The sense and anti-sense transcripts for each target sequence were incubated with RNase-free DN*aseI* (NEB, Ipswich, Massachusetts, United States) for 1 h at 37°C to remove residual template DNA. DN*aseI* digestion was stopped with0.5 M EDTA at 65°C for 10 min, followed by the phenol:chloroform-based extraction and ethanol-based precipitation of RNA transcripts. The single-stranded RNAs (ssRNAs), being self-complementary, were annealed in equimolar concentrations to generate respective ds*GFP* and ds*DES1* by initially incubating at 75°C for 10 min, and gradually cooling them down at room temperature. The dsRNAs were further run on 2% agarose gel with their corresponding ssRNA transcripts for integrity analysis and successful annealing. To prevent the formation of secondary structures, the ssRNAs were mixed with an RNA-loading dye, and denatured and chilled before loading into the wells. Next, ds*GFP* and ds*DES1* were purified using.a volume of 3 M sodium acetate (pH 5.2) and three volumes of chilled absolute ethanol. After overnight incubation at –20°C, the dsRNA was centrifuged in a Sigma 2–16 KL cooling-centrifuge at 13,000 rpm for 30 min. The precipitated pellets were washed twice with 70% ethanol, air-dried, and quantified with a UV spectrophotometer, and A_260/280_ ratio was measured (Supplementary Figure 2). A schematic representation of the synthesis of dsRNAs and *in vitro* and *in vivo* proof-of-concept experiments is provided in Figure 1.

### *In vitro* Feeding of Double-Stranded RNA to *Magnaporthe oryzae*

The *in vitro* treatment involved the co-incubation of wild type *M. oryzae* along with the purified dsRNA in both solid and liquid media. The fungus was grown on CM plates supplemented with 50 nM of ds*DES1* by inoculating hyphal block and incubating it for 9 days, Spores were isolated from the culture. Then, the spore suspension was diluted and spreaded on ds*DES1*-supplemented CM plates, following the procedure as published (Guo et al., 2019). An individual colony was further sub-cultured onto a 50 nM ds*DES1*-amended CM plate and allowed to grow at 28°C. This dsRNA-fed strain was used for phenotypic assays and transcript analyses of *MoDES1*, to study the effects of ds*DES1* on the wild type upon its uptake. The GFP fluorescence of *M. oryzaeGFP* was visualized by growing it both on sterile slides layered with YEG agar media, and on 24-well culture plates containing YEG broth. YEG agar was aliquoted, 2 ml each in a sterile centrifuge tube, cooled down, and supplemented with 5 nM ds*GFP*. The dsRNA-mixed YEG medium was layered onto sterile slides and allowed to solidify. A 10 μl drop of a 1 × 10^5^ spores/ml suspension was smeared along the length of the slide and incubated in the dark at 28°C for 2 days. In the liquid medium, a 10-μl drop of a 1 × 10^5^ spores/ml suspension was added to each well containing 1 ml of YEG broth and 1 ml of 5 nM ds*GFP* [dissolved in diethyl pyrocarbonate (DEPC)-water]. The culture plate was allowed to rotate at 45 rpm for mycelia formation and dsRNA-uptake. Conditions for the microscopic visualization of GFP fluorescence will be described later in the “Histochemical staining and microscopy” section. In order to mimic the oxidative stress in the environment, 1 mM H_2_O_2_ was added to the culture media, 30 min before RNA extraction from the dsRNA-fed and untreated axenic cultures. This was performed to elicit the transcription of *MoDES1*, which expresses in response to oxidative stress. The RNA was then used for the quantification of *MoDES1* transcripts in the treated and untreated fungi.

### Appressorium Penetration, Host Cell Colonization, and Infection Assay

An infection assay was performed on onion-peel, detached rice leaves and intact and abraded rice leaves, and 20 μl of a spore solution containing an equal number of spores (1 × 10^5^ spores/ml) was drop-inoculated on the sterile hydrophobic surface of the onion epidermis. Epidermal peels were placed on clean glass slides, kept inside moist petriplates, and incubated for 24 h at 28°C. Appressorium count was observed using a hemocytometer, and appressorium penetration was assessed following a published procedure (Chida and Sisler, 1987). The ease of cell-to-cell movement of invasive hyphae was determined by counting the infection units on onion epidermal cells and scoring them based on the penetration and branching pattern of invasive hyphae (IH) during *in planta* biotrophic growth. The percentage fraction of infection units showing 0, 1, and > 2-cell penetration was categorized considering 50 infection units in each case, with the experiment having three replicates; 10 μl drops of the 1 × 10^5^ spores/ml suspension [dissolved in 0.02% V/V Tween 20 (Merck, Bangalore, India) and 0.25% W/V gelatin] were spot-inoculated on the adaxial surface of detached leaves (Jia et al., 2003) and rice sheath (Kankanala et al., 2007) of 21 day-old plants. Mean lesion length and area were measured using the Image J software, whereas infection severity in terms of lesion densities per 5 cm^2^ leaf area (Liu et al., 2010) was evaluated by spray-inoculating 3-week-old plants (Akagi et al., 2015). Among other whole-plant infections, the leaves were either abraded and punch-inoculated (Park et al., 2016), or spot-inoculated. In both cases, 50 μl spore drops were inoculated on 4-week-old plant leaves, and drops were secured to leaf surface with adhesive tapes. The plants were maintained in a growth chamber with 27°C temperature and 90% relative humidity settings.

### Reactive Oxygen Species Sensitivity Test, Extracellular Enzyme Assay, and Protoplast Release Assay

Hyphal blocks of uniform size, from the 7-day old fungal culture were sub-cultured onto CM plates amended with 0, 3, and 7 mM H_2_O_2_, and incubated for 10 days at 28°C. ROS sensitivity was determined from the mean diameter of the growing culture at 5 and 10 dpi, and in all cases three biological replicates were maintained. Extracellular laccase activity was measured in solid CM media, supplemented with a 0.2 mM 2,2′-Azino-bis (3-ethylbenzthiazoline-6-sulfonic acid) (ABTS) substrate, while peroxidase activity was measured from 200 μg/ml Congo red (CR)-amended CM (Guo et al., 2019). The mean diameter of degradation halos was measured on 5 dpi and in triplicates. The laccase and peroxidase activity of the fungal culture filtrates was measured from the absorbance at 420 nm using 10 mM ABTS and 3 mM H_2_O_2_ (Chi et al., 2009). For the protoplast release assay, which is an indicator of cell wall integrity, mycelia were grown in CM, and protoplasts were isolated from 3-day-old culture (Chen et al., 2017). Observations were conducted after 90 min of lysis with a lysing enzyme, and the experiments were repeated thrice.

### *Ex vivo* and *in vivo* Treatment and Spraying of Double-Stranded RNA on Rice Leaves

For initial optimization, the detached leaves were sprayed locally with 50, 150, and 300 nM of ds*GFP* 24 h prior to *M. oryzaeGFP* inoculation. Later, for checking of systemic nature, 300 nM of ds*GFP* was sprayed in local and distal regions of sandpaper-abraded leaves, as described (Koch et al., 2016). The first and second (lower) leaves sprayed directly with dsRNA were considered as local regions, while the third and fourth leaves (upper) were considered as distal unsprayed parts. In case of infection on the punch-abraded leaves, 20 μl drops of 300 nM ds*DES1* were spotted on abraded areas of the leaves and were secured with adhesive tapes. The same abraded regions, 24 h later, were inoculated with 20-μl drops of the spore suspension *via* syringes, without disturbing the tapes. Similarly, dsRNA treatments were performed for the intact leaves in whole plants, but without abrasion. For the whole plant leaf infection assays in the experimental sets, sandpaper-abraded leaves were sprayed with purified 300 nM ds*DES1*, 0, 12, and 24 h before fungal spray inoculation. While in set I he dsRNA and spore suspension were mixed together and sprayed simultaneously (0 h), in set II and III, dsRNA was sprayed 12 and 24 h, respectively, prior to infection.

### Histochemical Staining and Microscopy

Fungal infection units were observed by staining the infected onion epidermal cells with a ready-to-use lactophenol blue (Sigma Aldrich, MI, United States) solution. The epidermal peels infected with conidia were stained with one drop of lactophenol blue for 15 min, destained with lactophenol until the excess stain got removed, and finally mounted with 60% glycerol before visualization. The infected rice sheaths were excised, and the epidermal layers of mid-vein were lactophenol-fixed and stained with 0.01% Aniline Blue (Chi et al., 2009) for the observation of callose plugs and secondary wall deposition in and the around primary infected cells. *In planta* ROS generation was visualized by staining the infected sheath epidermal cells at room temperature with a 1 mg/ml 3,3′-diaminobenzidine (DAB) (BioSB, CA, United States) staining solution prepared as per the instructions of the manufacturer. The sheaths were destained with a clearing solution (ethanol:acetic acid = 94:4 V/V) for 1 h and visualized under a differential interference contrast (DIC) filter. The infected cells and their neighboring cells were color-coded based on staining intensity, representing the level of H_2_O_2_ generation. The cell death assay involved overnight staining of the leaf sheaths with 0.01% trypan blue (HiMedia) at room temperature, followed by destaining in chloral hydrate for 24 h. The stained epidermal tissues were visualized in a bright field. All DIC and fluorescence images were taken using an Axio mRm camera coupled with a Zeiss Axio Imager A1 fluorescence microscope (Carl Zeiss, Göttingen, Germany). Fungal GFP fluorescence in the leaf lesions was observed under a fluorescein isothiocyanate (FITC) filter, and all the images were processed using the Zen Light Blue software. The mean intensity of GFP expressed by *M. oryzaeGFP* in the dsRNA- sprayed and unsprayed sets were documented considering leaves from at least three plants from each set in three independent experiments.

### RNA Isolation and Northern Blot Analysis

Total RNA was isolated from both the dsRNA-fed fungal strain and DEPC water-treated wild type fungus [4 days post treatment (dpt)], and dsRNA-sprayed and unsprayed infected rice leaf-lesions 3, 4, 5, and 7 dpt. The fungus and infected leaf biomass were crushed using liquid nitrogen and taken forward for RNA extraction using RNeasy Plant Mini Kit (Qiagen, Germany), as recommended by the manufacturers. The RNA was treated with RN*ase*-free DN*aseI* to remove traces of genomic DNA, assessed for quality and integrity, and further quantified by spectroscopy; 100 ng of RNA from local, distal-sprayed and unsprayed leaves was resolved on a 6% polyacrylamide gel (PAGE), and *in vitro*-transcribed ds*GFP* was kept as positive control. After the electro-transfer of the RNA onto a Ambion BrightStar positively charged nylon membrane, the membrane was hybridized with 150 ng of a non-radioactively labeled *GFP* probe (303 bp gel-purified PCR product). The primers used for PCR amplification of the *GFP* probe are listed in Supplementary Table 2. The labeling and detection of signals were performed using direct labeling reagents and CDP-Star (Amersham; GE Biosciences, United Kingdom) as per given instructions.

### Reverse Transcriptase-PCR and qRT-PCR

First-strand cDNA synthesis was performed by reverse transcribing a 1 μg template RNA with RevertAid H Minus M-MuLV Reverse Transcriptase (NEB, Ipswich, Massachusetts, United States). Reactions, 50 μl, were set up in a thermal cycler programmed at 42°C for 1 h, followed by termination of the reverse transcription at 70°C for 5 min. For transcript quantification by qRT-PCR, 2 μl of cDNA was taken as template in Power SYBR Green PCR Master Mix (Applied Biosystems, United Kingdom) consisting of gene-specific forward and reverse RT primers (Supplementary Table 2). Each reaction was carried out in triplicates, and the C_t_ values were normalized against the internal housekeeping control *MoACTIN* of the wild-type B157 strain. In the case of RT-PCR, 50 μl reactions were set up, each containing forward and reverse RT primers, deoxynucleotide triphosphates (dNTPs), 10 × reaction buffer, and 2.5 units of Taq DNA Polymerase (NEB, Ipswich, MA, United States). For molecular the analysis of fungal growth, the cDNA synthesized from the RNA extracted from infected leaf lesions were first diluted to different concentrations and calibrated using the rice housekeeping *OsACTIN*. Then, the amounts of cDNA corresponding to equal band intensity were used for the RT-PCR analysis of both the rice 25S rRNA and the fungal 28S rRNA. This was used to ensure that comparison of fungal growth was done in equal amounts of infected leaf tissue, from the experimental and control sets of rice plants. Standard PCR settings were maintained.

### Statistical Analysis

Each experiment was repeated at least three times independently. The statistical analyses were performed with the GraphPad Prism 8 (GraphPad, San Diego, CA, United States) software. Student’s *t-*test and pairwise comparison were performed by Tukey’s test with Bonferroni correction for the determination of significant outcomes. The alpha level was set at 0.05 in all cases.

### Biosafety Measures

All the relevant experiments from this study that involved the use of the phytopathogenic strain of *M. oryzae*, foliar spray of dsRNA, and infection assays of the rice plants were conducted following the biosafety norms as per Institutional Biosafety Committee (IBSC). To prevent environmental contamination through the dissemination of any of the above-mentioned agents, the experimental materials were handled under confined environment and laboratory conditions, restricted to growth chambers and the green house. Experimental wastes and unused materials were autoclaved and discarded in the end.

## Results

### BLAST Analysis of Sequences for Target Specificity Determination

Double-stranded RNA-mediated gene silencing is a target mRNA sequence-specific phenomenon. Hence, the *MoDES1* and *GFP* dsRNA sequences chosen as target were analyzed for selective specificity toward only the target gene and target organism. BLASTn analysis was done for the 303 bp and 300 bp target regions (Supplementary Figure 1) against *M. oryzae* and *O. sativa* genomic sequences. The *GFP* sequence showed significant similarity with neither the fungus nor the plant host. It only showed a 100% similarity with the *GFP* sequence from pCAMBIA1302. Similarly, the *MoDES1* target did not show any significant similarity with the *indica*, *japonica*, and *javanica* groups of rice. The BLAST analysis of *MoDES1* target across *M. oryzae* genome showed 100% sequence identity with only MGG_04163, which was the intended gene target. Three other *M. oryzae* strains, MZ5-1-6, LpKY97, and B71, showed 99.64% similarity with the query but had primary hosts other than rice (Supplementary Table 1). However, the chosen sequence for dsRNA generation would not lead to unspecific silencing in the B157 and 70–15 strains that are known to infect rice primarily. Ascomycetous fungi, such as *Chaetomium globosum*, *Podospora anserina*, *Neurospora crassa*, *Fusarium* sp., and *Botrytis cinerea*, have DES1 homologs and share a sequence similarity of greater than 44% with *MoDES1* (Chi et al., 2009). Sequences for the homologs were obtained from the Locus information and compared with the *MoDES1* target, and no significant similarity was observed (Supplementary Table 1). To this end, the target regions chosen from *MoDES1* and *GFP* were found to be specific only toward the intended *GFP* gene and MGG_04163, with no predictable off-targets in rice and *M. oryzae* used in the experiments.

### Double-Stranded RNA Synthesis and Dosage Optimization

**FIGURE 2:**
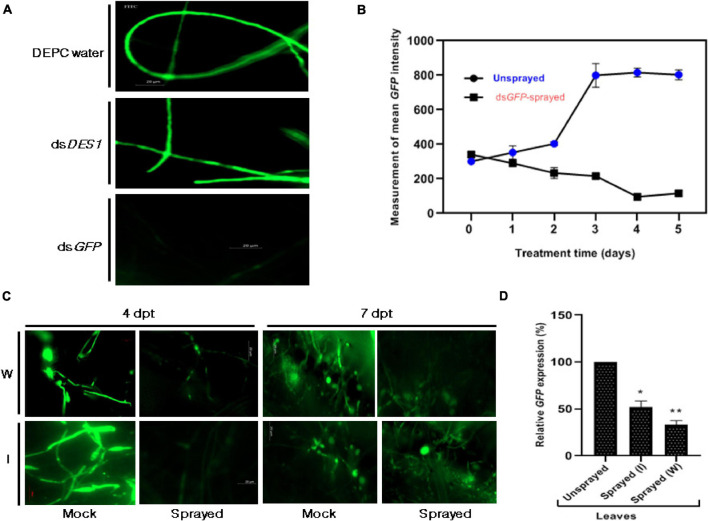
Effect of ds*GFP* on *M. oryzae GFP.* **(A)** Microscopic assessment of GFP intensity 3 dpt, among axenic cultures of *M. oryzae GFP* fed with and without ds*GFP*. While the culture that was not fed with any dsRNA (DEPC water- treated) was kept as the negative control, the culture that was fed with ds*DES1* (non-specific for *GFP*) served as the positive control. **(B)** Time-dependent fluorescence trend of *M. oryzaeGFP* grown on 300 nM ds*GFP*-sprayed leaves with respect to the unsprayed set, across 0–5 days. **(C)** Fluorescence of *M. oryzaeGFP* on wounded (W) and intact (I) rice leaves sprayed with ds*GFP*, 24 h prior to infection. GFP fluorescence was observed under an FITC filter of AxioCam mRm 0 [4 h post treatment (hpt)], 4, and 7 dpt, and fluorescence emitted by *M. oryzaeGFP* on Tris EDTA (TE)-sprayed leaves were kept as mock. TE was sprayed 24 h prior to infection, as replacement for ds*GFP*. Comparisons were made between the sets, at similar time points, with observations made from at least 10 leaves for each set. The experiment was repeated thrice, and the results were compared independently. **(D)** Relative abundance of *GFP* transcripts in dsRNA-sprayed wounded and intact leaves, with respect to TE-sprayed leaves, at 4 dpt. *OsACTIN* was used as internal control for normalization. The experiments were repeated thrice, each time with three biological replicates. Error bars represent standard deviation (*n* = 3), where * and ** denote statistical significance at *the P* ≤ 0.05 and *P* ≤ 0.01 levels, respectively.

A proof-of-concept experiment was performed with an *M. oryzae*-*GFP* reporter system for the demonstration of SIGS in rice. Similar to the previous reports, out of the many parameters that determine the efficacy of RNAi phenotype, the optimum dosage of dsRNA or asiRNA, abundance of the target gene, size of the target dsRNA, and sequence complementarity of the designed dsRNA and its delivery method are the most crucial (Bennett et al., 2020). The 303 bp and 300 bp target regions (Supplementary Figure 1) chosen from *GFP* and *MoDES1* were used, respectively, as templates for ds*GFP* and ds*DES1* generation. In each case, an average concentration of 30 μg/μl of purified dsRNA was obtained from one set of transcription and annealing reaction (Supplementary Figure 2). It was demonstrated that 50 nM of asiRNAs, when supplemented in media, could induce silencing of the target *M. oryzae* gene (Guo et al., 2019). Hence the efficiency of the chosen target ds*GFP* was assessed by *in vitro* treatment of *M*. *oryzaeGFP* spores with 50 nM of ds*GFP*. The microscopic evaluation of the mycelia (Figure 2A) revealed a 76% reduction in the fluorescence intensity of the dsRNA-treated mycelia compared with the DEPC water-treated controls 3 dpt. The specificity of the observed *GFP-*silencing phenotype was further confirmed using a positive control, where ds*DES1* was used for the *in vitro* treatment of *M*. *oryzaeGFP*. There was no observable reduction in fluorescence in this set, thereby attributing the formerly observed silencing effect to sequence-specific *GFP* knockdown *via* ds*GFP.* Since the effective dosage of dsRNA can often vary across organisms and delivery methods, optimization was performed by spraying 50, 150, and 300 nM of *in vitro* synthesized *GFP*-specific dsRNA (ds*GFP*) onto rice leaves, followed by *M*. *oryzae GFP* inoculation after 24 h. The silencing effect, in all the cases, was observed by measuring the mean intensity of GFP fluorescence in the inoculated *GFP*-transformant to optimize maximum silencing effect. It was observed that although the lower concentrations were able to induce silencing, the effect did not last beyond 36–48 h post treatment (data not shown). On the contrary, the effect of 300 nM ds*GFP* was perceived even after 4 days of application. During a 0–5 days time frame, the minimum intensity of GFP was observed 96 h post dsRNA treatment (hpt) or 72 h post inoculation (hpi) (Figure 2B), after which the effect of gene silencing started to reduce. Since 300 nM of the selected ds*GFP* was able to not only induce but sustain silencing for a longer period of time on the treated rice leaves, and considering that the abundance of our target pathogenicity gene *MoDES1* is unknown, the 300 nM dsRNA concentration was used for spraying and infection-related experiments to ensure effective RNAi.

### SIGS Is More Efficient and Sustained on Wounded Leaves

Ribonucleic acid molecules, being relatively more unstable than DNA or other biomolecules, have a limited longevity on plant surface. It has been shown that leaf cuticle can act as a barrier for uptake, hence, high-pressure spraying or abrading can ensure robust silencing (Dalakouras et al., 2016). Thus, we further wanted to check if removing physical barriers by sandpaper-mediated abrasion (Bennett et al., 2020), followed by spraying, would affect the longevity and efficiency of gene knockdown. Based on our previous observations on the detached leaf (Figure 2B), the 96 hpt time point was fixed for assessment and quantification of the silencing effect of ds*GFP* sprayed on both intact and wounded rice leaves. The microscopic observations revealed that the reduction in fluorescence beyond 4 dpt was more pronounced and sustained in the wounded ds*GFP*-sprayed sets (Figure 2C). Figure 2D further revealed a higher silencing efficiency in the wounded, dsRNA-sprayed leaves. The quantitative analyses showed that the relative abundance of the transcripts was relatively lower in the intact leaves, with 48% silencing, in contrast to the 67% knockdown observed in the case of the wounded leaves.

### Sprayed dsRNA Can Translocate and Assert Silencing in Distal Plant Parts

**FIGURE 3:**
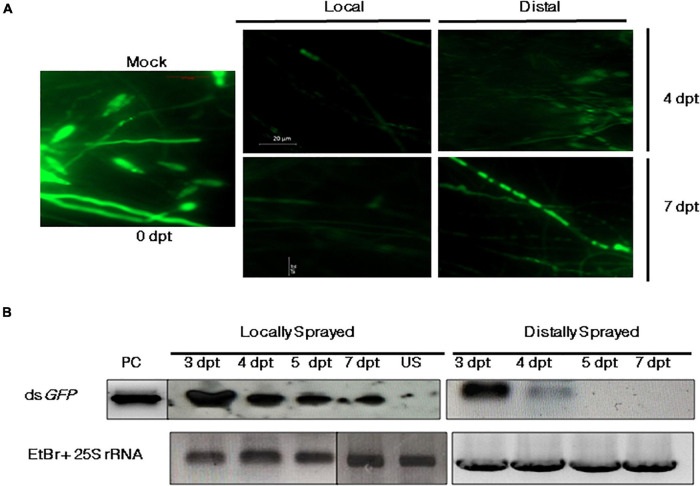
Systemic silencing activity of dsRNA and its accumulation in the local (sprayed) and distal (unsprayed) leaves. **(A)** Fluorescence microscopy of wounded *M. oryzaeGFP*-inoculated leaves that were either sprayed with either TE (mock) or ds*GFP* in local and distal regions. Mean fluorescence intensity of GFP from approximately five plants (10 lesions) of each set was monitored 4 and 7 dpt. **(B)** Northern blot profile for *GFP*-specific dsRNA. The longevity and accumulation of RNA signals were assessed 3, 4, 5, and 7 dpt on locally and distally sprayed leaves. The total RNA isolated from unsprayed (US) leaves was run as negative control. *In vitro*-synthesized and purified ds*GFP* was used as the positive control (PC), and rice 25S rRNA was kept as the loading control. Sense *GFP* probe with the same sequence as the parent ds*GFP* and PCR-purified rice 25S rRNA (primers listed in Supplementary Table 2) were used for hybridization.

The efficiency of SIGS largely relies on its systemic nature, i.e., the ability of RNA signals to travel from the sprayed region (local) to the unsprayed (distal) parts of the plant. To determine if the dsRNA can traverse to unsprayed regions, the detached leaves of 2-week-old rice seedlings were sprayed locally and distally with 300 nM of ds*GFP* or Tris-EDTA buffer (mock) and given a lag period of 24 h to facilitate the uptake of dsRNA by the leaf tissue. The leaves were then inoculated with 5 × 10^5^ spores/ml of *M. oryzaeGFP*. The microscope images showed relatively more intense fluorescence in the mock-treated infected leaves (Figure 3A) compared with the directly and distally ds*GFP*-treated leaves. Based on this finding and some previous reports on *Fusarium graminearum* (Koch et al., 2016) indicating silencing in distal parts of the leaves, whole plant infection experiments were conducted to explore the effectiveness of SIGS in *M. oryzae*. DsRNA specific to *GFP* were sprayed onto the wounded leaves to ensure effective silencing. The RNA extracted 4 dpt from the direct and distally sprayed leaves was assessed for the presence of spray-supplied 300 bp ds*GFP* by northern blotting. The results suggested the presence of ds*GFP* in both locally and distally treated regions. While in the directly sprayed parts dsRNA was detectable up to 7 dpt, in the unsprayed distal regions, dsRNA was detected up to 4 dpt. The accumulation pattern of ds*GFP* in the local and distal leaves of the dsRNA-sprayed plants was aligned with the time dependent reduction of silencing phenotype in the sprayed leaves, as shown in Figure 3A.

### Conidiation and ROS-Sensitivity Got Negatively Affected in ds*DES1*-Treated *M. oryzae*

**FIGURE 4:**
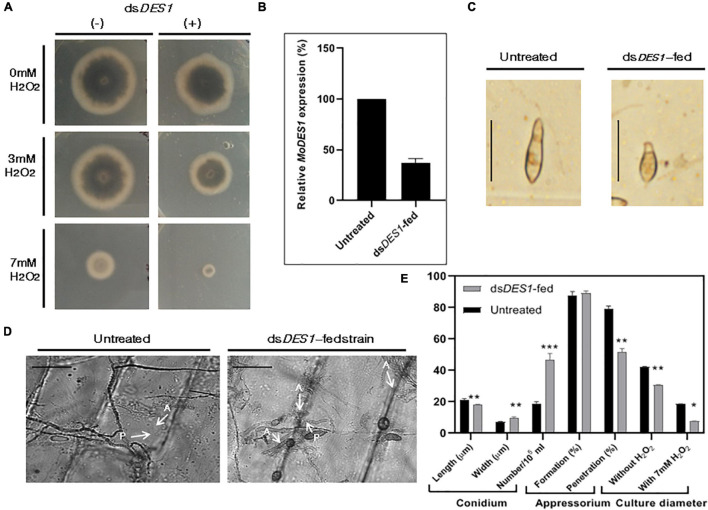
Assessment of ROS-scavenging activity, morphological traits, and target transcripts in the *in vitro* ds*DES1*- fed strain. **(A)** Reactive oxygen species (ROS) sensitivity assay of ds*DES1*-fed and untreated strains of wild-type B157. The ds*DES1*-fed and ds*DES1*-untreated strains were grown on CM plates with 0, 3, and 7 mM H_2_O_2_, and average growth diameter of the cultures was recorded from 5- to 10-day-old plates in triplicates. The experiment was repeated twice. **(B)** Relative *MoDES1* expression in the ds*DES1*-fed strain with respect to the untreated strain 72 hpt. The reactions were set up in triplicates, and *MoACTIN* was used as internal control. **(C)** The dominant morphological deformity in a major fraction of conidia isolated from the ds*DES1*-fed strain. For size reference, a 20-μm scale has been used. **(D)** Penetration potential of germinated appressoria among the untreated and ds*DES1*-fed strain, on onion epidermal peel. A 50-μm scale has been used for size- reference. Three replicates were used, and at least 50 conidia were assessed in each category. **(E)** Comparison of phenotypic parameters between untreated and ds*DES1*-fed strain. Error bars represent standard deviation, and *, **, *** denote statistical significance at the *P* ≤ 0.05, *P* ≤ 0.01, and *P* ≤ 0.001 level, respectively.

**FIGURE 5:**
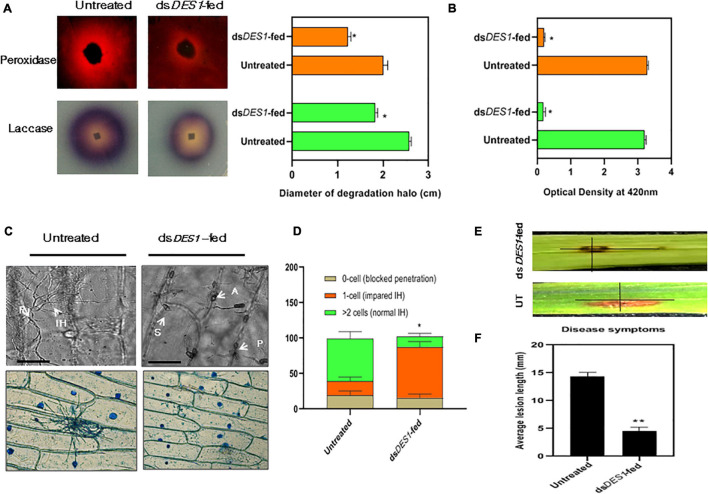
Extracellular enzyme activity and virulence potential of ds*DES1*-fed strain. **(A)** The extracellular laccase and peroxidase activity of the *in vitro*-ds*DES1*-fed strain on CM plates supplemented with ABTS and Congo red (CR), respectively. The average diameter of halo or discoloration indicator of substrate breakdown was measured in triplicates from 5-day-old plates. **(B)** Activities of laccases (green) and peroxidases (orange) in culture filtrates of the untreated and ds*DES1*-fed strain. **(C)** The upper panel indicates the 40 × -magnified differential interference contrast (DIC) image of onion peel infected with untreated and ds*DES1*-fed strain. The white arrows indicate the spore, S; appressoria, A; point of penetration, P; invasive hyphal, (IH) growth. The lower panel shows a 10 × -magnified bright field image of Lactophenol blue-stained fungal infection units grown on onion epidermal cells. This is indicative of the cell-to-cell movement of IH and host colonization. **(D)** Graphical representation of the fraction of infection units that showed differential ease of host-colonization, beyond the primary site of penetration. The potential for cell-to-cell movement of IH was color-coded based on the number of cells they penetrated (0– > 2). At least 50 different infection units were observed for each set, and the experiment was repeated thrice. **(E,F)** Spot inoculation of detached rice leaves with spores isolated from the *in vitro* ds*DES1*-fed strain and untreated wild-type strain. Average length of lesion was measured for each set with approximately 50 leaves (≈ 100 lesions). The error bars represent ± SD, and * and ** denote statistical significance at the *P* ≤ 0.05 and *P* ≤ 0.01 levels.

*MoDES1* is a novel gene encoding a hypothetical protein that was found to be involved in suppression of PTI in rice *via* host-derived ROS-scavenging activity. In a previous report, it has been functionally characterized and found to have homologs in other members of ascomycetes (Chi et al., 2009). Targeted gene silencing needs to be highly specific; hence, the selected target region of 300 bp is aligned with the genomic sequences of both rice and fungal members that possess a DES1 (Supplementary Table 1) homolog. The BLASTn and BLASTp results showed no significant homology, with no prediction of off-target effects. The *in vitro*-synthesized dsRNA was fed to the wild-type fungus, which after two generations of dsRNA treatment, showed a slight reduction in growth diameter, as compared with the untreated fungus (Figures 4A,E). The axenic cultures of the phenotypically distinguishable dsRNA-fed strain demonstrated an approximately 86% reduction in the *MoDES1* transcripts (Figure 4B). Although conidiation rate was significantly higher, the treated strain produced a majority of short and broad deformed conidia (Figures 4C,E). Besides, an impediment was noted in appressorial penetration with respect to the untreated strain, although the rate of appressorium production was comparable in both strains (Figures 4D,E). In order to check ROS-scavenging activity, the treated and untreated strains were grown in media supplemented with ≥2 mM H_2_O_2_. The dsRNA-fed strain showed a marked reduction in culture diameter in all the concentrations 5 and 10 dpi (Figure 4A and Supplementary Figure 3), depicting the apparent reduction in ROS scavenging potential. As per previous studies conducted on the *MoDES1* mutant, ROS sensitivity was due to impaired extra-cellular enzyme activity. Hence, enzymatic assays for laccases and peroxidases that are known to neutralize some free radicals were conducted. The dsRNA-fed strain that previously showed significant silencing of *MoDES1* was observed to demonstrate a pronounced reduction in the aforementioned enzymatic activities in both the solid and liquid media (Figures 5A,B). This corresponded with the reduced ROS-scavenging activity that has been seen previously. The cell-wall integrity test, in terms of protoplast release assay, did not show any significant defects in cell wall integrity as a result of *MoDES1* silencing (Supplementary Figure 4).

### Ds*DES1*-Feeding of *M. oryzae* Impaired Its Biotrophic Growth and Virulence

Based on previous studies, *M. oryzae* has been reported to use *DES1* as a suppressor of basal defense for survival within rice. Also, it has an active role in the extension of the IH inside host cell and its colonization, which is essential for pathogenicity. As a proof-of-concept for the *in vivo* whole plant spray experiments, the *in vitro* impacts of ds*DES1* on the *M. oryzae* wild-type strain were analyzed. Microscopy results of the onion peel assay showed that after 24 h, germination and IH development was defective in the ds*DES1*-fed strain as compared with the wild type. Most of the IH were thick, stout, and short, and confined to the primary host cell (Figures 5C,D), indicating impairment in the colonization of epidermal cells similar to the *DES1* mutant (Chi et al., 2009). Our *in vitro* dsRNA feeding experiment indicated that the strain of *M. oryzae* that was fed with ds*DES1* for two consecutive generations showed significant downregulation of *MoDES1*. Therefore, we further wanted to check if its virulence got affected because of dsRNA feeding. The detached leaves of 2-week-old rice seedlings were spot-inoculated with both the untreated and dsRNA-fed strains, and were observed for symptoms 5 dpi. While average lesion length in the case of the wild type was 12 mm, it was only 4.6 mm in the case of the leaves inoculated with the dsRNA-fed strain. Therefore, on an account of the silencing of *MoDES1*, there was almost a 62% reduction in lesion length (Figures 5E,F). As suggested by our previous results, this reduction in virulence could be attributed to the defects in IH and host colonization.

### Fungal ds*DES1* Uptake *via* Plant Cells Imparted Higher Disease Resistance

**FIGURE 6:**
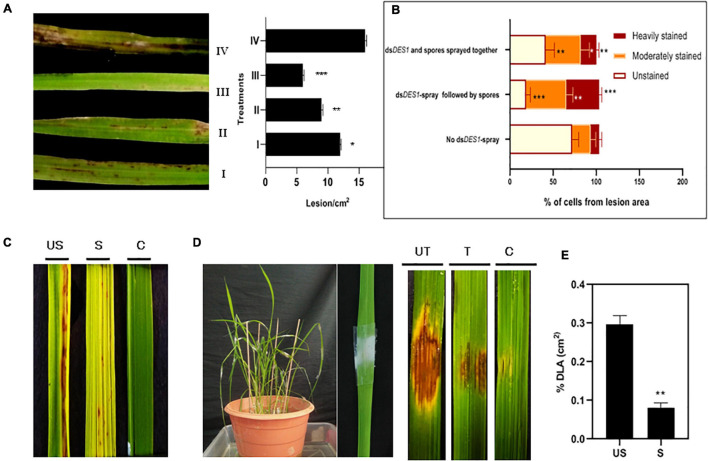
*In vivo* infection assay of ds*DES1*-sprayed plants. **(A)** The left panel shows spray-infected rice leaves that were sandpaper-abraded (to reduce the mechanical barrier) and sprayed with 300 nM ds*DES1* (III) 24 h prior, (II) 12 h prior, or (I) 0 h prior (spores and ds*DES1* were mixed together before spraying) to fungal inoculation. The unsprayed but fungus-inoculated set (IV) was used as control. The right panel indicates the relative infection severity for each treatment based on lesions/cm^2^. **(B)** DAB staining of lesions isolated from treatment sets I, III, and IV, representative of counter-infection host-derived ROS generation assay. The level of ROS generation was color-coded, and the fraction of cells representing each color was categorized. **(C)** Spray infection assay of ds*DES1*-sprayed (S) and unsprayed (US) leaves. Leaves that were neither dsRNA-sprayed nor spore-inoculated, were used as control **(C)**. **(D)** Punch inoculation-mediated infection assay for ds*DES1* drop-treated (24 h prior) rice leaves. Leaves that were simply punched and inoculated with Tween 20-Gelatin mixture without spores or ds*DES1* were kept as control. **(E)** Disease severity in ds*DES1*-sprayed and unsprayed leaves expressed in terms of% diseased leaf Area (DLA). For infection related experiments, observations were performed on at least 50 leaves, and% DLA was assessed using the Image J software. Error bars represent standard deviation (*n* = 50); where *, **, and *** denote statistical significance (Student’s *t*-test) at the *P* ≤ 0.05, *P* ≤ 0.01, and *P* ≤ 0.001 levels, respectively.

The dsRNA sprayed on the plant can be uptaken either directly by the fungus on the surface *or* via the plant cell post penetration. Since our results indicated that the silencing effect shows greater longevity in the wounded plants, it was assessed further if the fungus got silenced better *via* the plant cell. Four sets were kept, whereby no lag period was given in set I, and both the dsRNA and fungal spores were mixed together and sprayed. In set II, ds*DES1* was sprayed on rice leaves 12 h prior to spray inoculation with the fungal spores; in set III, dsRNA was sprayed 24 h before the fungal inoculation to facilitate dsRNA uptake *via* the plant cells. Set IV was the control group where no ds*DES1* was sprayed on the leaves before fungal infection. It was observed that in the plants where the dsRNA was sprayed 24 h before wild-type spray inoculation (set III), disease severity was around 60% less as compared with the set IV that was inoculated with wild type spores without any dsRNA treatment (set IV). On the contrary, when the wild-type spores were mixed together with dsRNA before spray-inoculation (set I), there was only a 25% reduction in symptoms, which was not significantly less than the untreated control (IV). The symptoms were milder in set II (where dsRNA and spores were sprayed 12 h apart) than in set I (Figure 6A). As studied by DAB staining of sheaths, the generation of *in planta* ROS indicated that the maximum number of heavily stained cells were observed in the plants that were sprayed with dsRNA first and then inoculated with fungal spores. In the plants where dsRNA and spores were mixed together and sprayed, the maximum number of cells showed medium staining or no staining, with lesser number of heavily stained cells (Figure 6B). This indicated lower levels of ROS-mediated resistance in the later. While in the first case, a lag period was given to ensure that the dsRNA would be uptaken by the host cell first, in the second case, the fungal spores were directly mixed with the dsRNA to ensure it takes up the dsRNA first. As our results indicated, the inhibitory effect of ds*DES1* was more pronounced, leading to higher disease resistance, when the dsRNA was sprayed prior to fungal infection, allowing for a lag period of at least 12 h. Taken together with the finding from wounded leaves, it can be assumed that processing of sprayed dsRNA by the plant might have an important role in amplifying the sRNA signals.

### *In planta* SIGS of *MoDES1* Confers Resistance Against *M. oryzae* via PTI Responses

**FIGURE 7:**
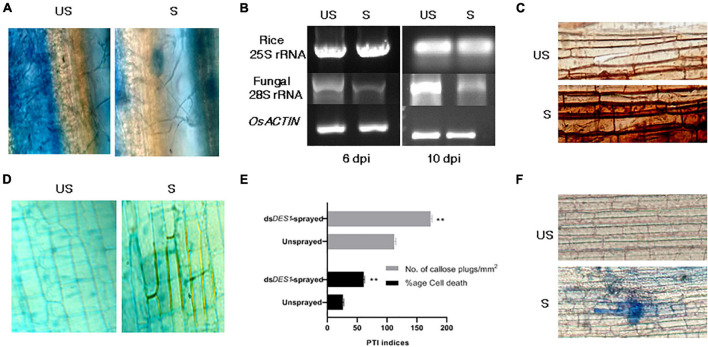
Evaluation of fungal growth and defense parameters of pathogen-associated molecular pattern (PAMP)-triggered immunity (PTI). **(A)** Qualitative assessment of fungal growth. Lactophenol blue staining of fungal mycelia observed in infection lesions from ds*DES1*-sprayed and unsprayed leaves. **(B)** Semi-quantitative reverse transcriptase PCR for *Mo*28S rRNA that is representative of fungal biomass in sprayed and unsprayed infected leaves. While *Os*25S rRNA was the growth control from rice that was used for comparison, *MoACTIN* served as the internal control. **(C)** DAB assay. Host-derived ROS generation in infected cells from ds*DES1*-sprayed and unsprayed leaf-sheaths was visualized by DAB staining. **(D)** Secondary wall deposition around primary infected cells and their neighbors to curb disease progression *via* aniline blue staining. **(E)** Callose plug formation (aniline blue staining) and percentage of cell death (trypan blue staining) in ds*DES1*-sprayed and unsprayed infected rice cells. **(F)** Trypan blue staining of sprayed and unsprayed sheath cells undergoing necrosis. The error bars represent standard deviation, and ** denotes statistical significance at the *P* ≤ 0.01 level.

After assessing the functionality of SIGS both *in vitro* and *in vivo*, we wanted to explore the efficiency of spray-induced downregulation of *MoDES1* in developing resistance against fungal blast disease in whole plants. For this, Leaves of the susceptible CO-43 rice variety were sprayed with ds*DES1* and inoculated with a 1 × 10^5^ spores/ml solution 24 h later (Figure 6C). The total RNA isolated 3 dpi from the dsRNA-sprayed leaves was analyzed for *MoDES1* transcripts, and it was found to have only 35% of relative abundance of what was observed in unsprayed plants. The ds*DES1-*sprayed leaves that were spray-inoculated showed a relatively lesser number of lesions/cm^2^ of infected leaves as compared with the control Tris-EDTA-treated (mock), spore-inoculated leaves. Similar results were obtained when the dsRNA-pre-treated leaves were punch- inoculated, whereby the area of lesions got drastically reduced (Figures 6D,E), with a notably reduced load of fungal biomass (Figure 7A). The fungal biomass was semi-quantitatively measured from the fungal specific 28S rRNA, with respect to the rice 25S rRNA, in both the sprayed and unsprayed infected leaves. When analyzed from the same amount of infected leaf tissue, the relative expression of 28S rRNA, both 6 and 8 dpi, was found to be lower in the sprayed set (Figure 7B). Since *MoDES1* is known to suppress PTI, we wanted to check if the resistance developed *via* sprayed ds*DES1* was due to innate host defense responses. The histochemical DAB and aniline blue staining of infected sheath sections showed that the host-derived ROS generation (Figure 7C), lignin deposition, and callose plug formation (Figures 7D,E) were more in the case of the dsRNA-sprayed sheaths. The microscopic analysis of dsRNA-sprayed infected sheaths showed that cell death response was at par with the resistance phenotype, as depicted by the trypan blue staining (Figure 7F). This corroborated with previous observations, as cell death is often a manifestation of mild HR, which is part of PTI in its mild form. The reduction in disease severity in the ds*DES1* spray-treated set could, hence, be attributed to the reduced fungal load observed in the infected leaves.

### *In planta* Silencing of *MoDES1* Led to Pathogenesis-Related Gene Induction in Double-Stranded RNA Treated Plants

**FIGURE 8:**
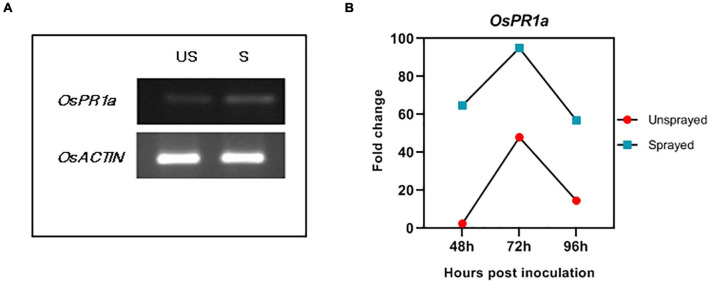
Expression of *OsPR1a*. **(A)** Semi-quantitative Reverse transcriptase PCR for rice defense- related *PR1a* in ds*DES1*-sprayed and unsprayed plants 72 hpi. **(B)** The relative fold change for *OsPR1a* expression in sprayed and unsprayed plants 48, 72, and 96 hpi, normalized against the house-keeping control *OsACTIN.* The reactions were set up in triplicates.

The phytohormone salicylic acid-mediated pathway in the hemibiotrophic rice-blast pathosystem is central to the defense armor of the plant. Apart from early PTI responses, such as ROS-burst and callose deposition, the induction of defense-related PR genes on account of increased endogenous SA levels is a crucial indicator of a robust SAR response. *OsPR1a* is one such marker gene that has generally been used to assess defense responses in rice. Hence, its expression levels were checked in the dsRNA-treated and untreated infected (punch-inoculated) plants 2, 3, and 4 dpi. The semi-quantitative qPCR (Figure 8A) showed that 3 dpi, the ds*DES1*-treated leaves showed a greater accumulation of *OsPR1a* transcripts. The quantitative real-time PCR exhibited that, in comparison with the internal housekeeping *OsACTIN*, 2 dpi, the untreated plants showed a 2.5-fold change in the *OsPR1a* transcript, while there was a 64.8-fold change in the treated plants. While 3 dpi, the *OsPR1a* mRNA had twofold upregulation in the ds*DES1*-treated plants, 4 dpi, the relative expression of *PR1a* was found to be almost threefold higher in the sprayed set with respect to their corresponding untreated sets (Figure 8B). These observations were in support of the argument that the mild necrotic responses in the sprayed plants were a result of SAR, which was a manifestation of the sustained upregulation of PR gene.

## Discussion

The prerequisite for the induction of RNAi is a dsRNA that bears complementarity with the gene targeted for silencing. Besides this, the efficiency of gene knockdown depends on additional factors such as size of the target region, basal expression of the gene, method of dsRNA delivery, and the system in which the induction is intended. Often, the dosage of dsRNA and its treatment technique for effective silencing vary among target plants, pathogens, and genes (based on their secondary siRNA signal amplification efficiency and target transcript abundance) (Das and Sherif, 2020). Our optimization studies, using a GFP-reporter system, revealed that the *in vitro* feeding of *M. oryzaeGFP* with 50 nM naked dsRNA through culture media could successfully induce the silencing of the target *GFP* transgene in the fungal mycelia (Figure 2A). We also found that the silencing effect of sprayed ds*GFP* on rice leaves could be more pronounced and lasted up to more than 4 dpt when the amount of dsRNA sprayed was increased to 300 nM (Figure 2B). This is in accordance with other reports where naked dsRNAs were sprayed against fungal pathogens to treat *Hordeum vulgare* (Koch et al., 2016), *Glycine max*, and *Triticum aestivum* (Gu et al., 2019). The uptake of the sprayed dsRNA by the plant tissues is crucial to assert gene silencing. However, there has been evidence in tobacco (Dalakouras et al., 2016) and Amaranthus leaves (Bennett et al., 2020) that the cuticle of leaves can act as a significant barrier against the uptake because of surface hydrophobicity. Hence, foliar spray experiments were carried out on both intact and sandpaper-abraded adaxial leaf surfaces (Huang et al., 2018; Guo et al., 2019). Our microscopic observations indicated a higher and sustained incidence of silencing in the wounded plants (Figure 2C), where a 67% reduction in *GFP* transcripts was seen as compared with 48% silencing in the intact set 4 dpt (Figure 2D). This could suggest that the silencing efficiency is more sustained in the abraded leaves because of the proper entry of the naked dsRNA and plausible replenishment of secondary siRNAs. On the contrary, the low silencing efficiency in the case of the intact leaves could either be due to a fraction of dsRNAs getting degraded by UV and environmental nucleases before reaching into the plant cell (Cagliari et al., 2018) nor lack of the efficient regeneration of sRNA signals, as found in *Fusarium asiaticum* (Song et al., 2018). Once sprayed on the leaf surface, the longevity and efficiency of functional naked dsRNAs can vary from plant to plant. While naked dsRNAs have shown silencing activity for only up to 7 days on tobacco leaves (Konakalla et al., 2016), a relatively longer period of stability of up to 28 days was observed in potato leaves (San Miguel and Scott, 2016). In the wounded and sprayed rice leaves, the reduced fluorescence phenotype of the *M. oryzaeGFP* was conspicuous even 7 dpt. Some reports have shown that topically applied dsRNA can move systemically across plant tissues to prevent pathogen colonization even in the unsprayed regions (Koch et al., 2016; Mitter et al., 2017; Song et al., 2018). Systemic silencing was also demonstrated (Hendrix et al., 2021), where it was hypothesized that 22-bp siRNAs could induce silencing in systemic regions of *Nicotiana benthamiana* by secondary siRNA generation. The key to a prolonged and robust silencing induced by sprayed dsRNA can be attributed to their systemic nature. When dsRNA was sprayed both distally and locally on detached leaves and whole plants, our microscopic results (Figure 3A) demonstrated systemic silencing, as reported previously in other pathosystems. The analyses of total RNA revealed the accumulation of ds*GFP* in both local (up to 7 dpt) and unsprayed regions (4 dpt) (Figure 3B). This supported the fact that the silencing of *GFP*, as visualized under the microscope, was specifically induced by the sprayed ds*GFP*, which got processed into siRNAs by dicer-like proteins. The higher relative abundance of dsRNA in both sprayed and unsprayed plant parts indicated that spraying of dsRNA can give a lasting effect to SIGS. With this, it can be concluded that the silencing effect in distal unsprayed areas can be attributed to the systemic nature of the dsRNA. This also corroborated the findings of Koch et al. (2016) in barley leaves and Konakalla et al. (2016) in tobacco leaves.

The efficiency of gene knockdown may depend on the length and region of the target sequence selected for dsRNA. Thus, having validated the functionality of SIGS in the rice-*M. oryzae* pathosystem, an *in vitro* proof-of-concept experiment was carried out to check the efficacy of the 300 bp target region selected for silencing *MoDES1*. It has been demonstrated previously (Guo et al., 2019) that *M. oryzae* is capable of uptaking artificial siRNAs (asiRNA) from media. Upon feeding the wild-type strain with ds*DES1* for two fungal generations, observable phenotypic changes were documented with a significant reduction in *MoDES1* transcripts. As revealed by the phenotypic and ROS sensitivity assays (Figure 4), the observations made in the dsRNA-fed strain were at par with the findings reported in *MoDES1* mutants (Chi et al., 2009). A majority of the conidia showing morphological deformity corroborated the partial loss of *MoDES1 via* knockdown. The role of ROS is instrumental in the progression of pathogen within its host, and in determining the fate of such biotic interactions (Shetty et al., 2007). Several reports including Samalova et al. (2014) claimed the quintessential role of host-derived ROS in defending *M. oryzae*, and the importance of ROS scavenging for successful virulence has been proved in *M. oryzae* and other fungi like *Ustilago maydis* (Molina and Kahmann, 2007). In *M. oryzae, MoDES1* is known to be involved in ROS scavenging, and the ds*DES1*-fed strains showed sensitivity toward media-derived H_2_O_2_ in a dose-dependent manner (Figure 4A). The role of pathogen-derived extracellular enzymes is critical to the dismutation of ROS generated during PTI (Apostol et al., 1989; Tanabe et al., 2011). Our results revealed that the laccase and peroxidase activities of the dsRNA-fed strain were significantly reduced (Figures 5A,B), and that these phenotypes were similar to that of *MoDES1* mutants. ROS generation in a host was correlated to disease progression in hemibiotrophic interaction based on the structure and dynamics of invasive hyphae (Tanabe et al., 2011). The ds*DES1*-fed strain showed a clear impediment in host cell penetration and cell-to-cell progression (Figures 5C,D), as attributed by their short and less profusely branched biotrophic growth. Additionally, it revealed an almost one-third reduction in the length of lesions produced by them with respect to the untreated wild type (Figures 5E,F). Hence, for the ds*DES1*-fed strain, the inability to detoxify host-derived ROS had obvious consequences on host cell colonization and virulence potential.

In SIGS, dsRNA can have two outcomes after being sprayed. It can either be uptaken by the fungus from the surface of the plant, or, it can be uptaken by the plant, processed into siRNAs, and then transferred to the interacting fungus *via* cross-kingdom RNA exchange (Song et al., 2018; Wang and Dean, 2020). In the intact leaves, the fungus takes time to penetrate into the host cell, and a larger fraction of RNA gets absorbed by the fungus readily (Koch et al., 2016) before making its way into the plant tissues. However, under such circumstances, a fraction of dsRNA also gets dried up and degraded on the surface itself, and only some of it gets taken up by the fungus. Primarily, better uptake of dsRNA and longevity of silencing efficiency were demonstrated when it was sprayed on abrading surface. Since the silencing phenotype was more in wounded or abraded plants, it led us to hypothesize that the sprayed dsRNA can assert RNAi more efficiently in the fungus if it gets maximally uptaken *via* the plant tissues. This hypothesis was later proved in spray-infection experiments (Figures 6A,B), where the disease resistance phenotype and *in planta* ROS generation, associated with the silencing of pathogenicity gene *MoDES1*, corroborated our assertion mentioned above. It was observed that relative disease resistance, correlated with the percentage silencing of *MoDES1*, was highest when the dsRNA was sprayed 24 h before the fungal inoculation. This indicated that SIGS was more efficient when the majority of dsRNA was uptaken by the fungus *via* the plant tissues. Studies on *Fusarium asiaticum* (Song et al., 2018) showed that the effect of the dsRNA uptaken by the fungus directly, is short lived, and that the intended silencing effect almost disappears after 9 h. This is attributed to the lack of secondary amplification of siRNAs in the fungus.

Based on existing reports on the cross-kingdom transfer of dsRNA and siRNAs (Wang et al., 2016; Dubrovina and Kiselev, 2019), it might also be possible that there is a cross-talk happening between rice and fungal systems. However, further research is required to elucidate this mechanism and the extent of involvement of rice and *M. oryzae*-silencing players. However, having evaluated the impacts of ds*DES1* on the wild type fungus *in vitro* and in detached rice leaves, the application of SIGS was extended to whole plants. As optimized previously, ds*DES1* was sprayed onto abraded leaves of whole rice plants, and 65% *in planta* silencing of *MoDES1*was achieved. Our final aim was to develop a PTI response robust enough for *M. oryzae* to succumb to the rice defense system, and the sprayed plants indeed showed stronger resistance against the pathogen (Figures 6C–E). Disease payoff in the form of size and density of lesions directly correlates with fungal growth and plant defense cascade. The leaves treated and sprayed with ds*DES1* manifested notably less severity (Figures 6E, 7A,B), and the histological assays of the leaf sheaths in the early biotrophic phase revealed the infection and defense-related structures at the *M. oryzae*-rice interface (Figures 7C–F). Higher percentage of host cells across infection units showed enhanced PTI responses through generation of ROS, callose deposition, and cell death. The attenuated virulence in the ds*DES1*-sprayed plants could be correlated with the reduced fungal biomass as indicated by the semi-quantitative expression of the fungal 28S rRNA with respect to the rice 25S rRNA (Figure 7B). As a part of PTI cascade, infection-related cell death and necrosis are generally associated with the relay of defense signals, leading to an eventual SAR in hemi-biotrophic interactions (Bari and Jones, 2009). In a resistant cultivar, prolonged induction of PR genes and generation of phytoalexins are a hallmark of active defense. *PR1* is a commonly used marker for the host defense response triggered, and its quantitative transcript analyses (Figures 8A,B) depicted that the dsRNA sprayed plants showed a higher level of PR gene expression, even 4 dpi, than the unsprayed controls.

**FIGURE 9:**
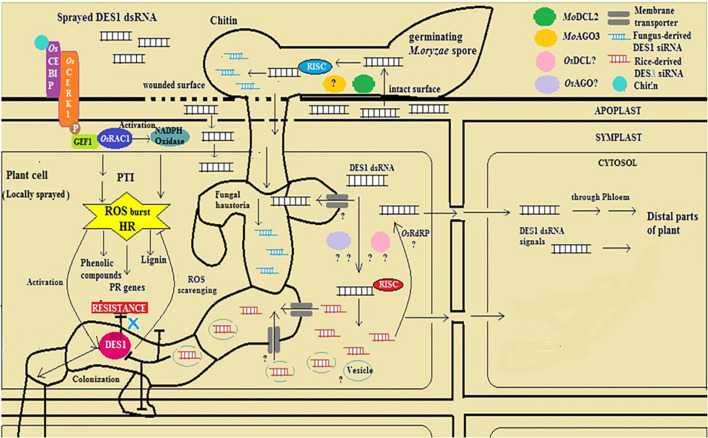
Diagrammatic representation of mode-of-action of ds*DES1* in conferring spray-induced gene silencing (SIGS)-mediated fungal blast resistance in rice.

Spray-induced gene silencing is a promising method of tackling the ever-evolving plant disease-causing fungi, exploiting the mechanisms of very fundamental processes, such as RNAi and cross-kingdom transfer of silencing RNA species. The topical application of naked dsRNA and nanoparticle-bound RNA-based pest and pathogen-control compounds backed up by sufficient biosafety evaluation is cost-effective and the environment-compatible future of RNAi technology for crop protection (Gebremichael et al., 2021; Taning et al., 2021). Besides, sprayed dsRNA can assert the knockdown of endogenous genes in plants, whereby physiological conditions and means of dsRNA application influence the efficacy of the silencing treatment (Kiselev et al., 2021). Based on the findings from this study and the reported mechanism of RNA exchange across plant and fungal cells, a possible mode of inductive action of dsRNA with reference to *MoDES1* in silencing-mediated defense elicitation in rice has been diagrammatically represented (Figure 9). To this end, our study validates that SIGS is an efficient strategy for targeted gene silencing and conferring disease resistance in the rice-blast pathosystem. Besides, this investigation sheds light on ways to optimally exploit SIGS as a crop-protection strategy and alternative to genetic modification. However, our investigation unravels possibilities for further research on the interaction between silencing players of host and pathogen, the extent of their involvement in secondary siRNA generation and silencing signal amplification, with reference to the rice-blast blast pathosystem, This relatively new and lesser-known dsRNA-based method can be better understood and exploited as an armament against several fungal phytopathogens in safeguarding their economically important host crops by means of addressing such general questions pertaining to its mechanistic details.

## Data Availability Statement

The original contributions presented in the study are included in the article/Supplementary Material, further inquiries can be directed to the corresponding author/s.

## Author Contributions

AS and SR-B conceived and designed the experiments, analyzed the data, and wrote the manuscript. AS performed the experiments. All authors read and approved the final version of the manuscript.

## Conflict of Interest

The authors declare that the research was conducted in the absence of any commercial or financial relationships that could be construed as a potential conflict of interest.

## Publisher’s Note

All claims expressed in this article are solely those of the authors and do not necessarily represent those of their affiliated organizations, or those of the publisher, the editors and the reviewers. Any product that may be evaluated in this article, or claim that may be made by its manufacturer, is not guaranteed or endorsed by the publisher.

## Footnotes

1
https://www.ncbi.nlm.nih.gov/nucleotide/

## Abbreviations

PAMP: Pathogen-associated molecular patterns
PRR: PAMP recognition receptor
ROS: Reactive oxygen species
HR: Hypersensitive response
PTI: PAMP-triggered immunity
ETI: Effector-triggered immunity
GFP: Green fluorescent protein
*GFP*: Gene-encoding green fluorescent protein
PR: Pathogenesis-related
*Os*CEBIP: *Oryza sativa* chitin elicitor-binding protein
*Os*CERK1: *Oryza sativa* chitin elicitor receptor kinase 1
HIGS: Host-induced gene silencing
SIGS: Spray-induced gene silencing
DCLs: Dicer like proteins
DES1: Defense suppressor 1 protein
*MoDES1*: Defense suppressor 1 gene
ds*GFP*: dsRNA specific to *GFP*
ds*DES1*: dsRNA specific to *MoDES1*
DAB: 3,3′-Diaminobenzidine
DIC: Differential interface contrast
FITC: Fluorescein isothiocyanate.
