# Supplementary material for: Spray-Induced Silencing of Pathogenicity Gene MoDES1 via Exogenous Double-Stranded RNA Can Confer Partial Resistance Against Fungal Blast in Rice
Source: Front Plant Sci. 2021 Nov 26;12:733129. doi: 10.3389/fpls.2021.733129 (PMC8662628; doi:10.3389/fpls.2021.733129)
Supplement: Supplementary file 1 [file Data_Sheet_1.pdf]

**(A) Strain**	**Accession**	**Query**	**%age nucleotide similarity**	**Natural host**
Pyricularia oryzae 70-15 uncharacterized protein (MGG_04163)	XM_003719617.1	100	100	*Oryza sativa* (Rice)
Pyricularia oryzae isolate MZ5-1-6	CP034209.1	100	99.64%	*Elusine coracana* (Finger millet)
Pyricularia oryzae strain LpKY97	CP050925.1	100	99.64%	*Lolium perenne* (Perennial ryegrass)
Pyricularia oryzae strain B71	CP060335.1	100	99.64%	*Triticum aestivum* (Wheat)
**(B) Species**	**Locus Name**	**E-value**	**Sequence identity**	**BLAST result**
Podospora anserina	Pa_6_2820	0	51.59%	No significant similarity
Chaetomium globosum	CHG04628.1	0	52.29%	No significant similarity
Neurospora crassa	NCU07707.2	0	50.18%	No significant similarity
Fusarium oxysporum	FOXG_08291	0	50.07%	No significant similarity

Fusarium graminearum	FGSG_09765.3	0	49.80%	No significant similarity
Botrytis cinerea	BC1G_03084.1	0	44.74%	No significant similarity

Supplementary Table 1. BLAST analysis of Sequence homology for checking specificity of *MoDES1* target sequence. (A) BLASTn analysis of *MoDES1* target sequence across *Magnaporthe oryzae* genome, showing hits obtained from different strains, their hosts and percentage sequence identity with maximum Query coverage for individual hits. (B) BLAST analysis of *MoDES1* target sequence across the DES1 homologs from Pezizomycetes members that showed >44% sequence similarity (Chi et al., 2009).

	**Name of Primers**	**Purpose**	**Sequence (5'→ 3')**
1	*GFP* FP	Probe generation	CACTGGAGTTGTCCCAATTC
2	*GFP* RP	Probe generation	CGTGTCTTGTAGTTCCCGTC
3	*GFP* FP Sense	Template preparation for *In vitro* transcription	GAAATTAATACGACTCACTATAGGGCACTGGAGTTGTCCCAATTC
4	*GFP* RP Sense	Template preparation for *In vitro* transcription	CGTGTCTTGTAGTTCCCGTC
5	*GFP* FP Anti-Sense	Template preparation for *In vitro* transcription	CACTGGAGTTGTCCCAATTC
6	*GFP* RP Anti-Sense	Template preparation for *In vitro* transcription	GAAATTAATACGACTCACTATAGGGCGTGTCTTGTAGTTCCCGTC
7	*MoDES1* FP	Template preparation for *In vitro*	GAAATTAATACGACTCACTATAGGGCGCTCGGGAAGCTTTT

	Sense	transcription	CAAC
8	*MoDES1* RP Sense	Template preparation for *In vitro* transcription	GACGTCTTCCTTGATGCTC
9	*MoDES1* FP Anti-Sense	Template preparation for *In vitro* transcription	CGCTCGGGAAGCTTTTCAAC
10	*MoDES1* RP Anti-Sense	Template preparation for *In vitro* transcription	<u>GAAATTAATACGACTCACTATAGGG</u>GACGTCTTCCTTGATGCTC
11	*MoACTIN* FP RT	Reverse Transcription PCR<br><br>Real Time qPCR	GCGGTTACACCTTCTCTACCAC
12	*MoACTIN* RP RT	Reverse Transcription PCR<br><br>Real Time qPCR	AGTCTGATCTCCTGCTCAAAG
13	*OsACTIN* FP RT	Reverse Transcription PCR<br><br>Real Time qPCR	TGCTATGTACGTCGCGCCATCCAG
14	*OsACTIN* RP RT	Reverse Transcription PCR<br><br>Real Time qPCR	AATGAGTAACCACGCTCCGTCA
15	*OsPR1a*FP RT	Reverse Transcription PCR<br><br>Real Time qPCR	TGCTATGCTACGTGTTTATG
16	*OsPR1a*RP RT	Reverse Transcription PCR<br><br>Real Time qPCR	AAATACGGCTGACAGTACAG
17	*GFP FP RT*	Reverse Transcription PCR	AGGAGCGCACCATCTTCTTC

		Real Time qPCR	
18	*GFP RP RT*	Reverse Transcription PCR<br><br>Real Time qPCR	TTGTACTCCAGCTTGTGCCC
19	*MoDES1 FP RT*	Reverse Transcription PCR<br><br>Real Time qPCR	CGCTCGGGAAGCTTTTCAAC
20	*MoDES1 RP RT*	Reverse Transcription PCR<br><br>Real Time qPCR	GACGTCTTCCTTGATGCTC
21	*25SrDNA FP RT*	Reverse Transcription PCR<br><br>Real Time qPCR	ACATACTACTGCGTTCCGGC
22	*25SrDNA RP RT*	Reverse Transcription PCR<br><br>Real Time qPCR	CCATGCGCACAGGATTTCAG
23	*28SrDNA FP RT*	Reverse Transcription PCR<br><br>Real Time qPCR	TACGAGAGGAACCGCTCATTCAGATAATTA
24	*28SrDNA RP RT*	Reverse Transcription PCR<br><br>Real Time qPCR	TCAGCAGATCGTAACGATAAAGCTACTC

Supplementary Table 2. The list of primers used in this study. The underlined sequences represent the T7 promoter sequence that was added to the primers for generation of Sense (S) and Anti-sense (AS) templates. These templates were used for *In vitro* transcription.

Target region from *GFP* sequence (amplified from pCAMBIA1302) :

CACTGGAGTTGTCCCAATTCTTGTTGAATTAGATGGTGATGTTAATGGGCACAAATTTTCTGTCAGTGGAGAGGGTGAAGGTGA
TGCAACATACGGAAAACTTACCCTTAAATTTATTTGCACTACTGGAAAACTACCTGTTCCGTGGCCAACACTTGTCACTACTTTCT
CTTATGGTGTTCAATGCTTTTCAAGATACCCAGATCATATGAAGCGGCACGACTTCTTCAAGAGCGCCATGCCTGAGGGATACG
TGCAGGAGAGGACCATCTTCTTCAAGGACGACGGGAACTACAAGACACG --

Target region from *MoDES1* sequence (amplified from *M.oryzae* b157 strain) :

GCTCGGGAAGCTTTTCAACCTGACGGCCGGCAACAATGGCGACGACGCCACAGGTGCACAGGATCAAAGCTCAACATCTC
CCTCACAGCTGATTTCAACCCTCGAGTCCGTCCAGGAGGACATACATACCCGCAACTTGCTATTCCCCGACGCCGAGTCTCT
GTTTCAGCACCGCAACGATCAGGTCTTTCCACTTTCGGCCGGTCCAGCTCTTCCGTCGACGTCGACCGCTGTCAACTCTTTT
GACTATGACTGCGACGTCTTCCTTGATGCTC --

Supplementary Figure 1: Target regions from *GFP* and *MoDES1* chosen as template for *in vitro* transcription with Sense and Anti-sense pair of primers (in red font-colour).

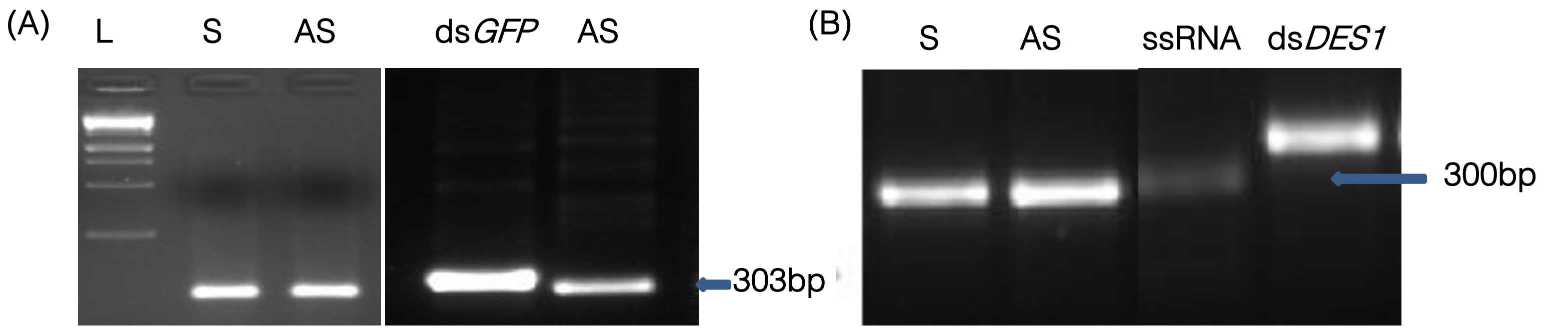

Supplementary Figure 2. S (sense) and AS (anti-sense) ssRNA and purified dsRNA after DN*aseI* treatment. (A) The left gel is a 2% agarose gel showing ssRNA transcripts of *GFP*, and the right gel is also a 2% agarose gel, showing purified ds*GFP*. (B) The left gel is a 2% agarose gel showing ssRNA transcripts of *MoDES1*, and the right gel is a 2% agarose gel, showing purified ds*DES1*. The dsRNA appears a little higher than their corresponding ssRNAs as it has undergone annealing.

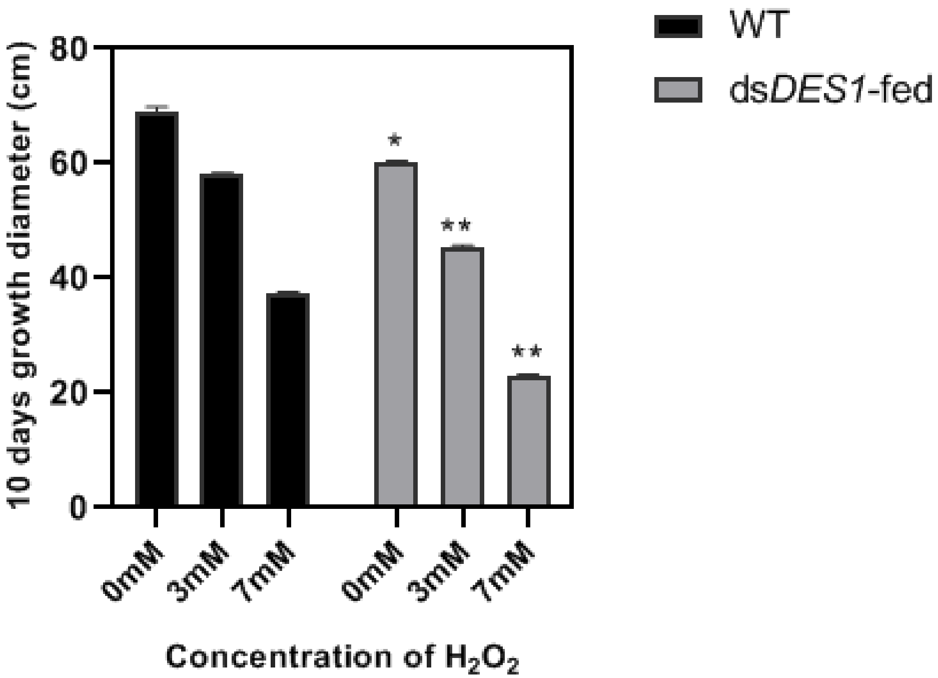

Supplementary Figure 3. ROS sensitivity test. Growth of WT and ds*DES1*–fed strains on H_2O_2-amended complete media (CM).

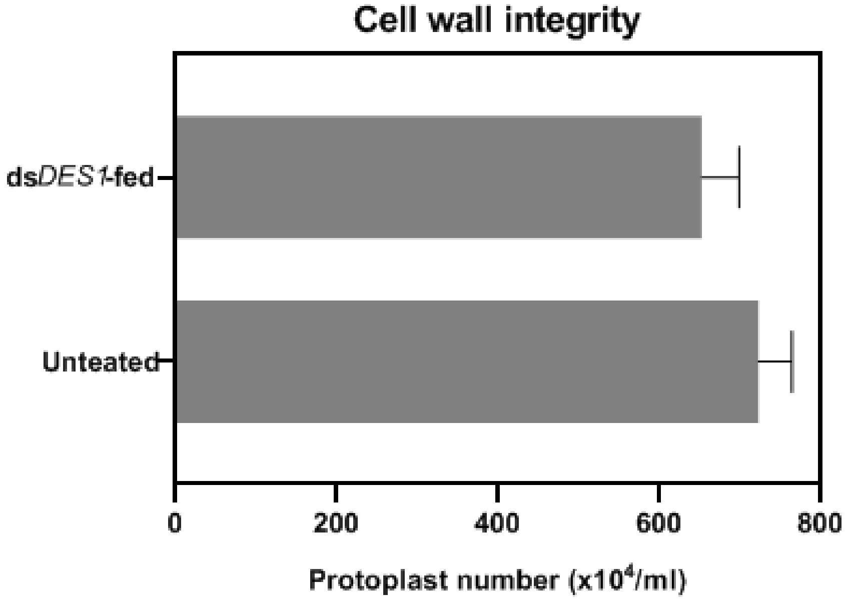

Supplementary Figure 4. Protoplast assay for checking cell-wall integrity of WT and ds*DES1*–fed strains at 90 mins time point, post-lysis.
